# OLFML2B Is a Robust Prognostic Biomarker in Bladder Cancer Through Genome-Wide Screening: A Study Based on Seven Cohorts

**DOI:** 10.3389/fonc.2021.650678

**Published:** 2021-11-15

**Authors:** Jiaxing Lin, Xiao Xu, Tianren Li, Jihang Yao, Meng Yu, Yuyan Zhu, Dan Sun

**Affiliations:** ^1^ Department of Urology, The First Hospital of China Medical University, Shenyang, China; ^2^ Department of Pediatric Intensive Care Unit, The Shengjing Hospital of China Medical University, Shenyang, China; ^3^ Department of Gynaecology, The First Hospital of China Medical University, Shenyang, China; ^4^ Department of Reproductive Biology and Transgenic Animal, China Medical University, Shenyang, China

**Keywords:** bladder cancer, Kaplan-Meier, macrophage, marker, prognosis

## Abstract

**Background:**

Bladder cancer lacks useful and robust prognostic markers to stratify patients at risk. Our study is to identify a robust prognostic marker for bladder cancer.

**Methods:**

The transcriptome and clinical data of bladder cancer were downloaded from multiple databases. We searched for genes with robust prognosis by Kaplan-Meier analysis of the whole genome. CIBERSORT and TIMER algorithm was used to calculate the degree of immune cell infiltration.

**Results:**

We identified OLFML2B as a robust prognostic marker for bladder cancer in five cohorts. Kaplan-Meier analysis showed that patients with a high level of OLFML2B expression had a poor prognosis. The expression of OLFML2B increased with the increase of stage and grade. We found that patients with high expression of OLFML2B still had a poor prognosis in two small bladder cancer cohorts. OLFML2B also has the prognostic ability in ten other tumors, and the prognosis is poor in high expression. The correlation analysis between OLFML2B and immune cells showed that it was positively correlated with the degree of macrophage infiltration and highly co-expressed with tumor-associated macrophage markers. Finally, the Wound-healing assay and Colony formation assay results showed that the migration and proliferation ability of bladder cancer cell lines decreased after the knockdown of OLFML2B.

**Conclusions:**

In summary, OLFML2B is a robust risk prognostic marker, and it can help patients with bladder cancer improve individualized treatment.

## Background

Bladder cancer is the ninth most frequently diagnosed disease worldwide. In 2020, new cases of bladder cancer accounted for 3% of all cancers, and new deaths accounted for 2.1% of all cancers (1). Smoking is the most important risk factor (2). Without systematic optimal treatment, the recurrence and fatality rates are very high (3). Although surgery and other treatments have been greatly improved in the past three decades, the clinical outcome of bladder cancer has not improved substantially (4). Although molecular-related treatment guidelines have become an essential pillar of many other cancer therapies, the management of bladder cancer depends on stage and grade, neither of which accurately reflect the risk of an individual patient (5). Many studies have shown that molecular changes in bladder cancer promote tumor progression (6), suggesting that the exploration of molecular markers will be the key to successful individualized treatment.

Bladder cancer has been at the forefront of developing biomarkers, monitoring their recurrence, and predicting clinical outcomes (7). Due to the development of sequencing technology, there are many markers and molecular subtypes of bladder cancer tissue for risk stratification and treatment options (8). In the lund-subtype based on gene expression profile (9), urobasal A subtype showed a very good prognosis, while urobasal B and SCC-like prognosis was the worst. Studies have shown that the DNA damage repair gene ERCC2 can predict the response to cisplatin-based neoadjuvant chemotherapy (10). In addition, the emergence of new detection techniques and methods has accelerated the accurate treatment of bladder cancer. At present, a variety of urine biomarkers has been developed to identify bladder cancer and prognostic risk stratification, including DNA mutation, protein determination, and RNA expression level (11). Circulating tumor cells have been proposed as a prognostic tool to improve the clinical management of bladder patients (12). These new markers and techniques will become potential prognostic markers for bladder cancer. However, bladder cancer shows a high degree of cellular and molecular heterogeneity, and some markers are not sensitive enough or do not have universal application. Mining prognostic molecular markers with robust prognostic functions and the combined use of various markers are essential tasks in bladder cancer research.

In this study, we downloaded bladder cancer transcriptome data from multiple gene expression databases to identify a robust prognostic marker of bladder cancer, and to explore the carcinogenic mechanism of bio-marker in terms of genome and tumor immunity. Finally, we compared the prognostic ability of reported gene prognostic markers in multiple cohorts.

## Materials and Methods

### Data Download and Processing

We searched The Cancer Genome Atlas (TCGA, https://cancergenome.nih.gov/) and Gene Expression Omnibus (GEO, http://www.ncbi.nlm.nih.gov/geo/) databases for bladder cancer transcriptome data. The original RNA sequencing and clinical data of bladder urothelial carcinoma (BLCA, n = 412) were downloaded from TCGA. The raw RNA sequencing and clinical data of GSE13507 (n = 165) (13), GSE32548 (n = 146) (14), and GSE32894 (n = 308) (9) were obtained from GEO. The normalized RNA expression matrix and clinical data of E-MTAB-1803 (n = 85) (15) were downloaded from ArrayExpress (https://www.ebi.ac.uk/arrayexpress/). They are bladder cancer cohorts containing transcriptome data and clinical follow-up information, the more specific cohort information is shown in **Table S1**. Then, the R package “edge” was used to standardize RNA expression matrix of TCGA-BLCA, GSE13507, GSE32548, and GSE32894. And the expression data of E-MTAB-1803 was standardize by R package “affy”. Finally, we selected patients with complete RNA sequencing, survival time, and survival status to proceed to the next step of the analysis. The specific clinical information of the rest of the sample is shown in **Table 1** and the cohort. The subtype data of bladder cancer were obtained from UCSC Xena (http://xena.ucsc.edu/).

**Table 1:** Basic clinical information of five cohorts.

Clinical factors	TCGA_BLCA		GSE13507		E-MTAB-1803		GSE32548		GSE32894	
	n=403	%	n=165	%	n=73	%	n=130	%	n=224	%
Age										
≦60	107	26.55	46	27.88	22	30.13	27	20.77	46	20.54
>60	296	73.45	119	72.12	51	69.86	103	79.23	178	79.46
Gender										
Male	298	73.95	135	81.82	62	84.93	99	76.15	163	72.77
Female	105	26.05	30	18.18	11	15.07	31	23.85	61	27.23
T stage										
<T2	4	0.99	104	63.03	0	0	91	70	173	77.23
≧T2	366	94.29	61	36.97	73	100	38	29.23	51	22.77
Grade (WHO 2004)										
Low	20	4.96	105	63.64	–	–	–	–	–	–
High	380	94.29	60	36.36	–	–	–	–	–	–
Grade (WHO 1999)										
G1	–	–	–	–	0	0	15	11.54	45	20.09
G2	–	–	–	–	4	5.48	40	30.77	84	37.50
G3	–	–	–	–	69	94.52	75	57.69	93	41.52
Vital status										
Alive	248	61.54	96	58.18	30	41.10	105	80.77	199	88.84
Dead	155	38.46	69	41.82	43	58.90	25	19.23	25	11.16
Follow-up (mean ± SD, year)										
	2.10 ± 2.23		3.98 ± 3.10		2.40 ± 2.44		4.14 ± 2.38		3.28 ± 2.10	

SD, Standard Deviation.

### Kaplan–Meier Analysis and Cox Regression Analysis

In tumors, the Kaplan-Meier survival curve is a commonly used tool to study the relationship between drug efficacy, clinical characteristics, gene expression, and disease prognosis. The R package “survival” can be used to draw Kaplan-Meier curves. When the p-value is less than 0.05, the two curves can be distinguished, indicating differences in survival conditions between the two groups. We use the cyclic algorithm to calculate each gene of the whole genome in turn. Our analysis method belongs to multiple hypothesis testing. Because the amount of data is too large, false-positive will inevitably occur. Generally speaking, it is necessary to correct the p-value. However, we finally set that the genes with a p-value < 0.05 are statistically significant, rather than false discovery rate (FDR). Because there are many limiting factors and shortcomings in correcting genomic p-value, routine correction is not recommended (16). The reported articles on large-scale univariate survival analysis have not corrected the p-value (17, 18). The function “res.cat” of the R package “survival” was used to identify the best cut-off values. The clinicopathological factors were analyzed by univariate and multivariate Cox regression analysis by the R package “survival”.

### Receiver Operating Characteristic (ROC) Curves

We used the R package “survivalROC” to plot the ROC curves of overall survival rates in 1/3/5 years and to calculate the area under the curve (AUC). It is generally believed that AUC >0.5 suggests good predictive ability; higher values suggest more accurate predictions.

### Genome Alteration and DNA Methylation

CBioPortal for Cancer Genomics is an open-access open-source resource (https://www.cbioportal.org) for interactive exploration of multiple cancer genomics data sets (19, 20). We used this tool to query genome alterations of the gene. UALCAN is a comprehensive interactive Web resource for analyzing cancer OMICS data (http://ualcan.path.uab.edu/index.html) (21). This tool can be used to query the DNA methylation of genes in normal and tumor tissues.

### Verification of Gene Prognostic Ability

The normalized RNA expression matrix and clinical data of GSE31684 (n = 93) (22), and GSE48075 (n = 73) (23) were obtained from Gene Ontology Consortium (GEO, http://www.ncbi.nlm.nih.gov/geo/). The online tool “Sangerbox” is a bioinformatics data integration platform that can analyze the pan-cancer of a single gene.

### Compare the Prognostic Ability of Genes

In the review of “Molecular Prognostication in Bladder Cancer” (5), the prognostic markers at the molecular level of the bladder were summarized in detail. We extracted prognostic markers at the expression level (**Table S2**), and then performed a Kaplan–Meier analysis of these genes in multiple cohorts.

### Co-Expression and Enrichment Analysis

Co-expression of genes can be queried using the web tool “cBioPortal” (19). The Metabase (http://metascape.org) was used for pathway and process enrichment analysis (24). The enrichment analysis included “KEGG Pathway, GO Biological Processes, Reactome Gene Sets, Canonical Pathways and CORUM” to evaluate the potential biological functions and pathways of these genes. We used the default screening criteria for the database: when p < 0.01, q-value < 0.05, with a minimum count of 3 and enrichment factor > 1.5. And we selected the first 20 items that meet the criteria for display.

### Exploration of Immune Characteristics

The R package “CIBERSORT” was used to calculate the content of 22 kinds of immune cells in the sample (25). This is a deconvolution algorithm that can estimate the cell composition of complex tissues based on standardized gene expression data. This method can quantify the abundance of specific cell types. We used the R package “ggstatsplot” and “ggplot2” to calculate and visualize the Spearman correlations. The TIMER Web server is a comprehensive resource (https://cistrome.shinyapps.io/timer/#tab-1050-3) for systematic analysis of immune infiltration of various cancer types (26). The CellMarker database provides a comprehensive range of cell markers (http://biocc.hrbmu.edu.cn/CellMarker/) in human and mouse tissues (27), which is helpful to explore the immune cells of concern to this present study.

### Cells and Culture

Human bladder cancer (BC)-derived T24 cells were obtained from the Chinese Academy of Sciences Cell Bank (China). Cells were maintained in RPMI 1640 medium (Gibco, USA) supplemented with 10% heat-inactivated fetal bovine serum (Gibco). Cells were cultured in incubators with humidified atmospheres of 5% CO2 and 95% air at 37°C.

### Transfection

According to the manufacturer’s instructions, cells were transfected using Lipofectamine^®^ 3000 (Invitrogen; Thermo Fisher Scientific, Inc., USA). Negative control siRNA, siRNA1, and siRNA2 against OLFML2B (Suzhou GenePharma Co., Ltd., China) were introduced into T24 cells at a final concentration of 10 nM, respectively. Forty-eight hours after transfection, the expression level of OLFML2B was confirmed by real-time PCR.

### Patients and Tissue Samples

Eleven pairs of BC tissues and their corresponding adjacent non-cancer tissues were collected from patients who underwent operation from Jun. 2019 to Dec. 2019 at the First Hospital of China Medical University. The adjacent normal tissues were collected at a distance of more than 5 cm from tumors. All tissues were processed for the histological examination. The Research Ethics Committee approved the present study of China Medical University, and all patients signed the written informed consent (**Table S3**).

### RNA Isolation and Quantitative Real-Time RT-PCR (qRT-PCR)

Total RNA, including RNA from cultured cells and frozen bladder tissues, was extracted using a miRNeasy Mini kit (Qiagen; GmbH), according to the manufacturer’s protocol. cDNA was synthesized using a Prime Script RT Master Mix kit (Takara Biotechnology Co., Ltd.; cat. no. RR360A). PCR reactions were performed using the SYBR Premix Ex Taq™ kit (cat. no. RR420A). ACTIN was used as internal controls. The sequences of the primers were as follows: OLFML2B (forward): 5’-GACAAGGTCAAGGCTATGTCTG-3’; OLFML2B (reverse): 5’-TGGTTTCCACGGTATAGAAGTCT-3’; ACTIN (forward): 5’-ACTTAGTTGCGTTACACCCTT-3’, ACTIN (reverse): 5’-GTCACCTTCACCGTTCCA-3’.

### Colony Formation Assay

T24 cells were seeded into six−well plates at a density of 1x103 cells/well in RPMI 1640 medium supplemented with 10% heat−inactivated FBS. At one week post−seeding, images were acquired using a light microscope (magnification, x40). The number of viable colonies was defined as >50 cells/colony. Results were quantified using ImageJ 1.51v software (National Institutes of Health).

### Wound-Healing Assay

T24 cells were seeded into six−well plates at the density of 6x105/well, maintained at 37˚C overnight, and transfected with negative control siRNA, siRNA1, and siRNA2 against OLFML2B. When the culture had reached ~90% confluency, the cell layer was scratched with a sterile plastic tip. The cell layer was then immediately washed twice with PBS and cultured in serum−free RPMI 1640 medium at 37˚C. At 0 and 12 h time points following scratch, wound healing was measured. The closure area of the wound was calculated as follows: Migration area (%) = (A0−A12)/A0x100, where A0 represents the area of initial wound area and A12 represents the remaining area of the wound after 12 h. The areas were quantified using ImageJ 1.51v software.

### Statistical Analysis

All statistical analyses are carried out using R software (Rx64 3.5.1). All R packages are obtained from CRAN (https://cran.r-project.org) and BioConductor (http://www.bioconductor.org). The “Wilcox test” was used to compare the two groups. The “Kruskal test” was used to compare the multiple groups. We used the R package “beeswarm” and “ggstatsplot” to visualize the comparison result. P < 0.05 means statistical significance.

## Results

### Genome-Wide Screening of Prognostic Markers

**Figure 1:**
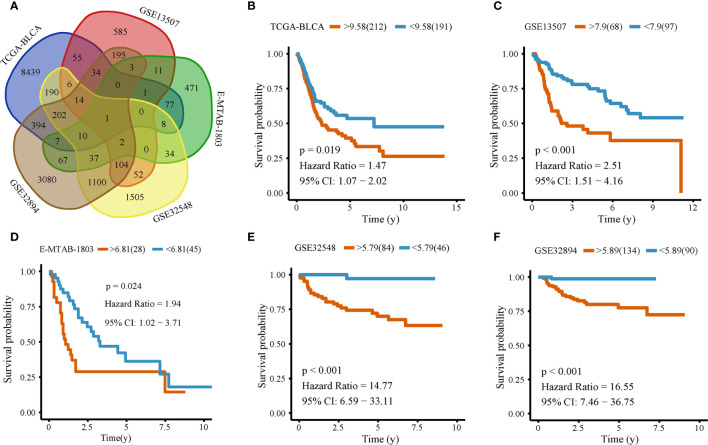
Kaplan–Meier analysis of OLFML2B. Venn picture of the Kaplan-Meier analysis result **(A)**. Kaplan–Meier analysis of OLFML2B expression in TCGA-BLCA **(B)**, GSE13507 **(C)**, E-MTAB-1803 **(D)**, GSE32548 **(E)**, GSE32894 **(F)**. The red line represents the high expression group; the blue line represents the low expression group, P < 0.05 indicates that it is statistically significant. CI represents the confidence interval.

We downloaded the transcriptome and clinical data of bladder cancer from several databases. After data preprocessing, we left 995 samples. They are TCGA_BLCA (N = 403), GSE13507 (N = 165), E-MTAB-1803 (N = 73), GSE32548 (N = 130), and GSE32894 (N = 224). All genes of the five cohorts were taken Kaplan-Meier analysis. In the analysis of each gene, the patients were divided into two groups according to the gene expression’s median value. The survival time of the two groups was analyzed by the log-rank test, it is a kind of single factor analysis. We use the cyclic algorithm to do a Kaplan-Meier analysis of each gene in each cohort, so the analysis between each cohort and each gene is independent. When p < 0.05, we define it as a prognostic gene (**Table S4**). We selected and intersected the prognostic genes in five cohorts and found that only OLFML2B was their common prognostic gene (**Figure 1A**, **Table S5**). To show the best predictive ability of the gene, we used the optimal cutoff value of each cohort to group separately, redraw the Kaplan-Meier curve of TCGA_BLCA (P = 0.019; **Figure 1B**), GSE13507 (P < 0.001; **Figure 1C**), E-MTAB-1803 (P = 0.024; **Figure 1D**), GSE32548 (P < 0.001; **Figure 1E**) and GSE32894 (P < 0.001; **Figure 1F**). All five cohorts consistently showed that the prognosis of the group with high expression of OLFML2B was worse than that of the group with low expression of OLFML2B.

### Both ROC Curve and Cox Analysis Indicate That OLFML2B Has a Robust Prognostic Ability

**Figure 2:**
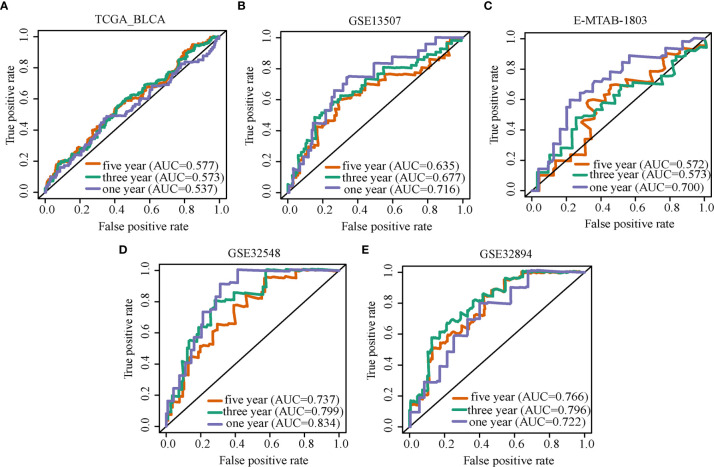
ROC curve of OLFML2B. ROC curve analysis of OLFML2B expression in TCGA-BLCA **(A)**, GSE13507 **(B)**, E-MTAB-1803 **(C)**, GSE32548 **(D)**, GSE32894 **(E)**. The purple line represents the ROC curve of the one-year overall survival rate, the green line represents three years, and the red line represents five years.

ROC curves and AUC values were used to evaluate the diagnostic value of markers. Overall survival ROC curves of OLFML2B expression were drawn in five cohorts, and the AUC corresponding to each curve was calculated. The AUC of the TCGA-BLCA cohort in 1/3/5 was 0.537, 0.573, 0.577 (**Figure 2A**); that of GSE13507 was 0.716, 0.677, 0.635 (**Figure 2B**); that of E-MTAB-1803 was 0.700, 0.573, 0.572 (**Figure 2C**); that of GSE32548 was 0.834, 0.799, 0.737 (**Figure 2D**); and that of GSE32894 was 0.722, 0.796, 0.766 (**Figure 2E**). All AUC values greater than 0.5 indicate that OLFML2B has prediction ability in all five cohorts. Of these, OLFML2B predicted the overall survival rate of one year better in the cohorts of GSE13507, E-MTAB-1803, GSE32548, and GSE32894 (AUC ≥ 0.700). Univariate Cox analysis showed that OLFML2B was statistically significant in TCGA-BLCA, GSE13507, GSE32548 and GSE32894 cohorts (p < 0.05, **Table 2**). Multivariate Cox analysis showed that OLFML2B only had statistical significance in E-MTAB-1803 and GSE32894 cohorts (p < 0.05, **Table 2**).

**Table 2:** Univariate and multivariate COX regression analysis of clinical factors and OLFML2B on overall survival of patients with bladder cancer.

Variables	Univariate analysis		Multivariate analysis	
	HR (95% CI)	P	HR (95% CI)	P
TCGA-BLCA				
Age	1.04(1.02-1.06)	**3.69E-05**	1.04(1.02-1.06)	**1.66E-04**
Gender	0.85(0.59-1.22)	3.74E-01	0.84(0.59-1.21)	3.60E-01
Grade	26257554.67(0-Inf)	9.95E-01	3539739.69(0-Inf)	9.92E-01
T stage	1.76(1.38-2.25)	**6.40E-06**	1.77(1.37-2.29)	**1.61E-05**
OLFML2B	1.15(1.04-1.27)	**6.37E-03**	1.08(0.98-1.2)	1.22E-01
GSE13507				
Age	1.07(1.04-1.1)	**4.53E-08**	1.07(1.04-1.1)	**3.07E-07**
Gender	0.64(0.36-1.14)	1.29E-01	0.78(0.42-1.43)	4.19E-01
Grade	2.74(1.69-4.43)	**4.00E-05**	0.94(0.51-1.71)	8.33E-01
T stage	2.05(1.64-2.58)	**5.06E-10**	2.09(1.57-2.79)	**4.78E-07**
OLFML2B	1.65(1.24-2.2)	**5.46E-04**	1.03(0.72-1.48)	8.67E-01
E-MTAB-1803				
Age	0.99(0.96-1.02)	6.83E-01	1(0.97-1.03)	8.35E-01
Gender	1.08(0.45-2.59)	8.63E-01	1.8(0.7-4.64)	2.23E-01
Grade	0.88(0.31-2.49)	8.12E-01	1.08(0.38-3.11)	8.84E-01
T stage	2.92(1.88-4.53)	**1.74E-06**	3.04(1.92-4.83)	**2.34E-06**
OLFML2B	1.31(0.97-1.75)	7.39E-02	1.4(1.02-1.92)	**3.90E-02**
GSE32548				
Age	1.04(0.99-1.08)	9.34E-02	1.05(1.01-1.1)	**2.82E-02**
Gender	1.29(0.48-3.43)	6.13E-01	1.37(0.49-3.84)	5.50E-01
Grade	2.26(1.07-4.77)	3.26E-02	0.62(0.21-1.83)	3.85E-01
T stage	3.53(1.89-6.6)	**7.42E-05**	4.22(1.69-10.5)	**1.98E-03**
OLFML2B	2.19(1.48-3.24)	**9.12E-05**	1.47(0.9-2.41)	1.26E-01
GSE32894				
Age	0.98(0.95-1.01)	1.79E-01	0.96(0.91-1.01)	8.44E-02
Gender	1.47(0.55-3.93)	4.45E-01	1.13(0.42-3.06)	8.14E-01
Grade	7.59(2.45-23.52)	**4.45E-04**	5.8(1.82-18.45)	**2.91E-03**
T stage	0.98(0.62-1.56)	9.42E-01	0.96(0.58-1.6)	8.87E-01
OLFML2B	2.06(1.49-2.86)	**1.56E-05**	1.62(1.11-2.35)	**1.27E-02**

HR, hazard ratio; CI, confidence interval; Inf, infinity.

Bold means p < 0.05.

### OLFML2B Promotes the Clinical Progress of Bladder Cancer

**Figure 3:**
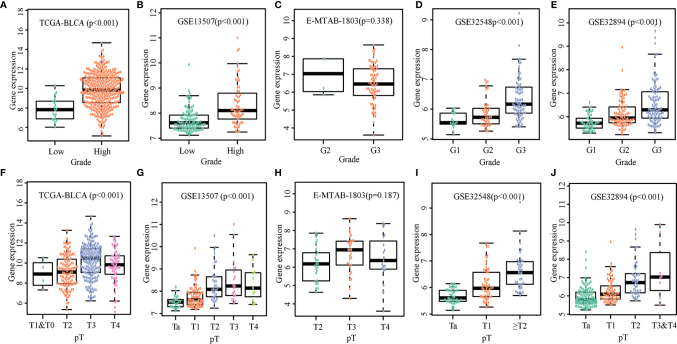
Expression levels of OLFML2B in various grades and T stages. The expression scatters plot of OLFML2B under different grades in five cohorts are shown in **(A–E)**. The expression scatters plot of OLFML2B under different T stages in five cohorts are shown in **(F–J)**. P < 0.05 indicates that the expression is statistically significant, and the expression is different in different clinical states.

We compared expression levels of OLFML2B in various grades and T stages. The wilcox.test was used to compare the two groups; the Kruskal.test was used for multi-group comparisons. There were significant differences in gene expression with different grades and T stages among TCGA-BLCA (**Figures 3A, F**), GSE13507 (**Figures 3B, G**), GSE32548 (**Figures 3D, I**), and GSE32894 (**Figures 3E, J**), except E-MTAB-1803 (**Figures 3C, H**). With the increase of grades and T stages, the expression of OLFML2B also increased.

### Other Genomic Explorations of OLFML2B

**Figure 4:**
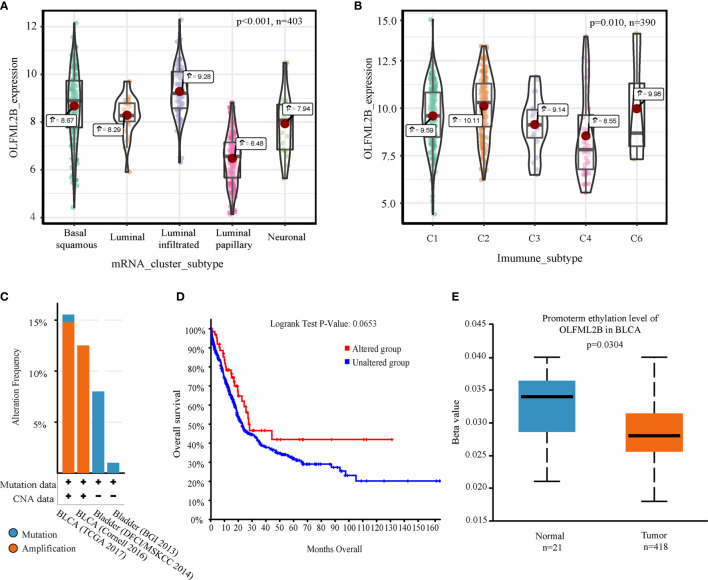
Comprehensive analysis of OLFML2B. **(A)** Expression levels of five mRNA types which contained basal squamous, luminal, Luminal infiltrated, luminal papillary, and neuronal subtypes, respectively. **(B)** The expression levels of the five immunophenotypes were C1 (wound healing), C2 (IFN-gamma dominant), C3 (inflammatory), C4 (lymphocyte depleted), C6 (TGF-b dominant). **(C)** A bar chart of the proportion of genomic changes in four cohorts of OLFML2B. **(D)** According to the Genome alteration of OLFML2B, the patients were divided into two groups for Kaplan–Meier analysis. **(E)** Comparison of methylation levels of OLFML2B in normal and tumor tissues. P < 0.05 indicates that it is statistically significant.

Kruskal-test was used to compare the difference of OLFML2B expression under different subtypes. Expression levels of OLFML2B differed depending on the mRNA cluster (P < 0.001; **Figure 4A**). Expression levels were highest in the luminal-infiltrated cluster and were lowest in the luminal-papillary cluster. OLFML2B expression levels also differed by immune subtype (p = 0.010; **Figure 4B**), with the highest expression in C2 (IFN-gamma dominant) and the lowest in C4 (lymphocyte-depleted) subtype. We used cBioPortal to explore genome alterations in OLFML2B and found that it had a large genome alteration in several cohorts. OLFML2B showed more than 12% amplification in both “BLCA (TCGA 2017)” and “BLCA (Cornrll 2016)” data sets, and more than 7% mutation in “Bladder (DFCI/MSKCC 2014)” data sets (**Figure 4C**). According to the existence of OLFML2B genomic changes, the samples were divided into two groups for Kaplan–Meier analysis. The prognosis of the altered group was better than that of the unaltered group, although there was no significant difference (P = 0.0653; **Figure 4D**). The methylation status of OLFML2B in bladder cancer was queried using the web tool UALCAN. We found that OLFML2B was hypomethylated in bladder cancer (P = 0.0304; **Figure 4E**).

### OLFML2B Was Validated Successfully in Two Small Cohorts

**Figure 5:**
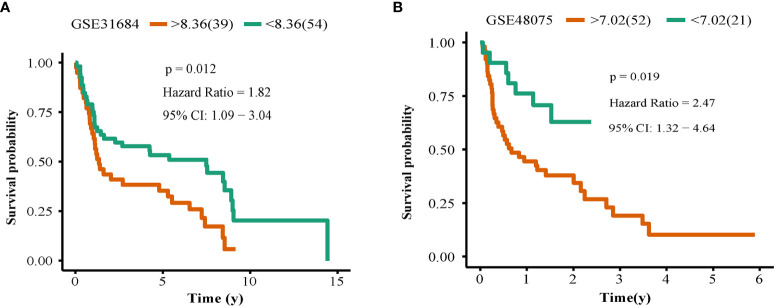
Survival verification. The Kaplan–Meier curve of OLFML2B in the GSE31686 cohort **(A)**, GSE48075 cohort **(B)**. The red line represents the high expression group, and the green line represents the low expression group, P < 0.05 indicates that it is statistically significant.

We found that prognosis of patients with high expression levels of OLFML2B was worse than those with low expression levels in E-MTAB-1803 (N = 93; P = 0.012; **Figure 5A**) and GSE4807 (N = 73; P = 0.019; **Figure 5B**), suggesting that OLFML2B was successfully verified in these two smaller cohorts. This result strongly validates the ability of OLFML2B to predict prognosis.

### OLFML2B Showed Poor Prognosis Under High Expression in Other 10 Kinds of Cancer

To explore whether OLFML2B has the same prognostic ability in other cancers, the Kaplan-Meier analysis of OLFML2B in 33 cancers can be queried in the web tool “SangerBox.” We found that OLFML2B had statistical significance in adrenocortical carcinoma, bladder urothelial carcinoma, breast invasive carcinoma, kidney renal clear cell carcinoma, kidney renal papillary cell carcinoma, lung adenocarcinoma, ovarian serous cystadenocarcinoma, sarcoma, stomach adenocarcinoma, thyroid carcinoma, and uveal melanoma (P < 0.05; **Supplementary Figure S1**). And all of them were poor prognosis in the high expression group.

### Comparison of Published Prognostic Genes for Bladder Cancer

**Figure 6:**
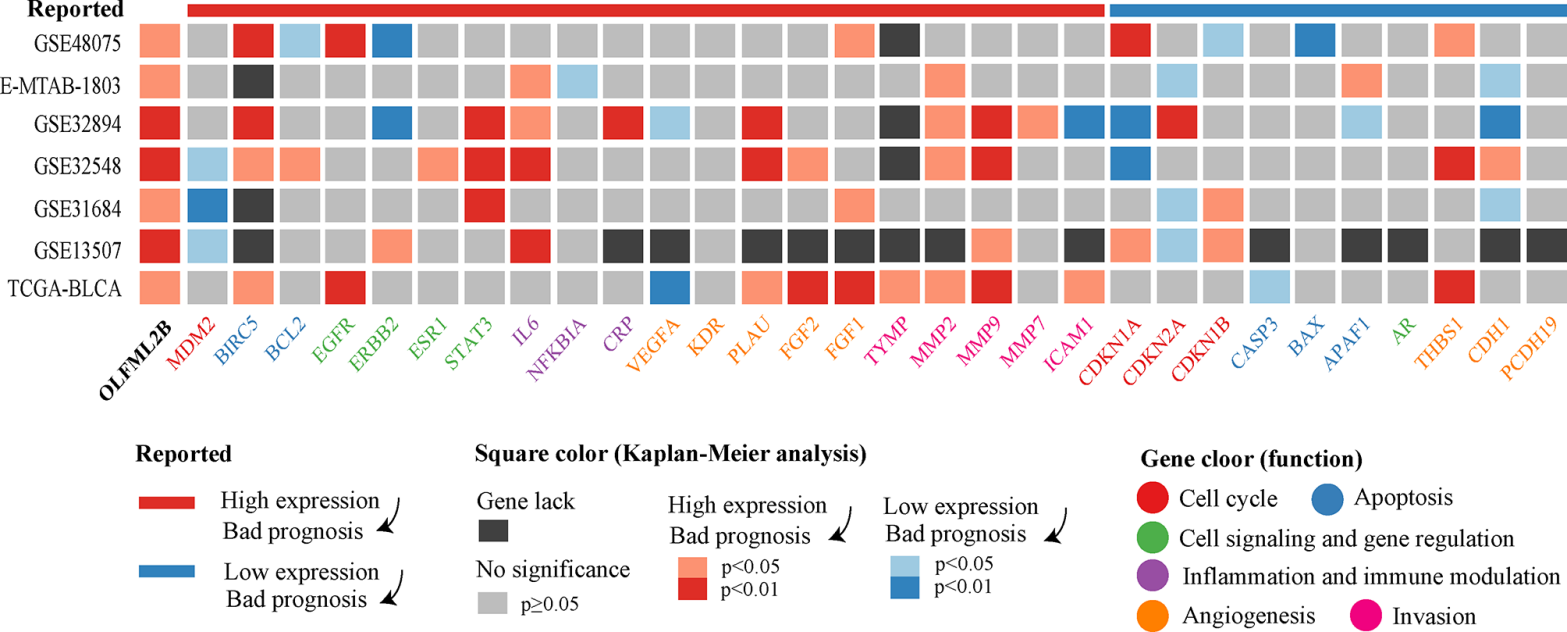
Kaplan–Meier analysis results of OLFML2B and 30 markers. The uppermost band represents the effect of gene expression on prognosis in the reported articles. The color of the square represents the results of the Kaplan–Meier analysis. Gene names have different colors, indicating the primary function of genes.

According to the Mitra’s review, there are 30 prognostic markers for bladder cancer in terms of expression level. We refer to genes predicting poor prognosis at high expression levels as risk genes, and the genes predicting poor prognosis under low gene expression as protective genes. There were 20 risk genes and ten protective genes. The 30 markers were analyzed using the Kaplan–Meier method in seven cohorts (**Figure 6**). Among the 20 risk genes, BIRC5, IL6, MMP2, and MMP9 had the strongest prognostic ability and were successfully predicted in four different cohorts. Among the ten protective genes, CDKN2A and CDH1 had stronger prognostic ability and were successfully predicted in three different cohorts. Among these genes, we found genes that are opposed to the previously reported results, such as MDM2, VEGFA, and THBS1. We counted the results of these two groups of genes to compare their overall prognostic ability. Among the risk genes, 28.57% of results were successful, and 7.14% of results were opposite to the reported results. Among the protective genes, 18.46% of results were successful, and 15.38% of results were opposite to the reported results. Based these two indicators, the success rate of risk gene verification was higher and more stable than that of protective gene verification.

### Enrichment Analysis of Co-Expressed Genes to Explore the Potential Function of OLFML2B

**Figure 7:**
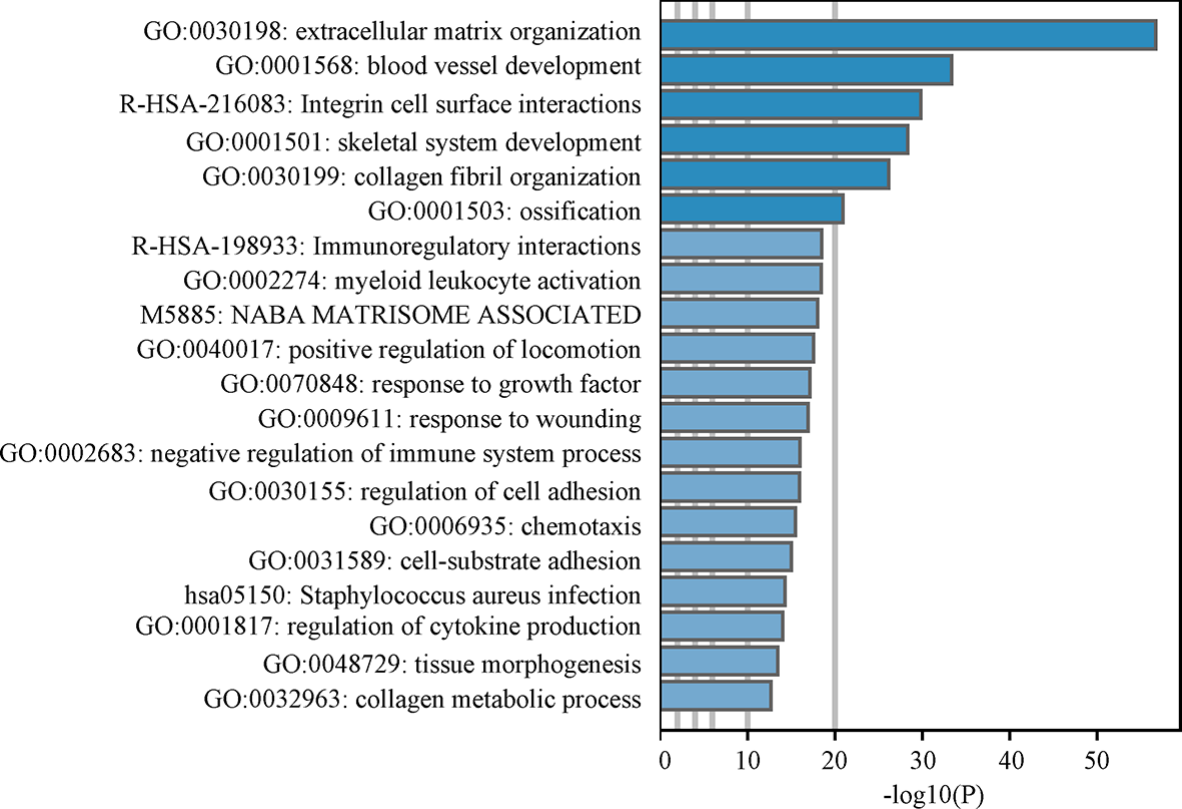
Enrichment analysis. The bar chart is the results of Metascape enrichment analysis. Larger the-log10 (P) implies greater significance.

In the “BLCA (TCGA 2017)” cohort of web tool “cBioPortal”, 561 genes were found to be positively related to OLFML2B with Spearman correlation more than 0.6. These genes were inputted into the web tool “Metascape” for enrichment analysis. The first three terms of enrichment were “extracellular matrix organization”, “blood vessel development”, and “integrin cell surface interactions” (**Figure 7**), all of which are related to the tumor microenvironment.

### The Expression of OLFML2B Is Proportional to the Infiltration Level of Macrophage

**Figure 8:**
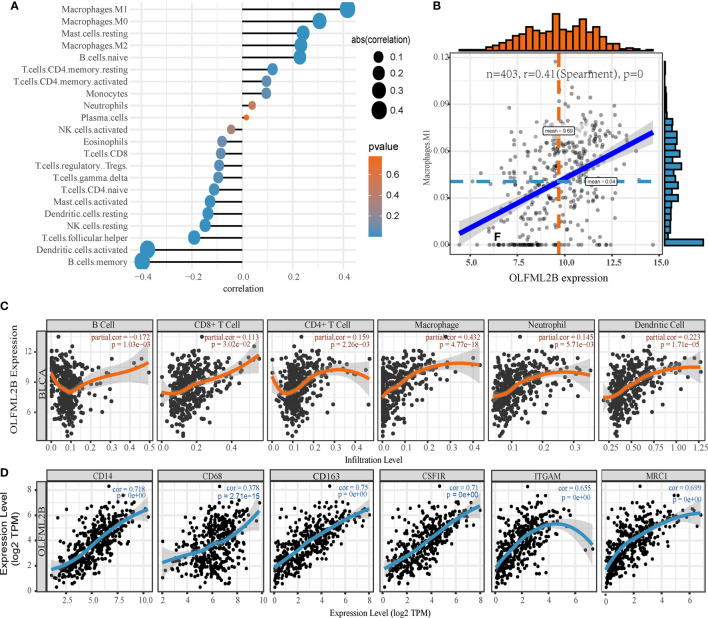
Exploration of immune characteristics of OLFML2B. **(A)** The Spearman correlation between OLFML2B and 22 kinds of immune cells. **(B)** The correlation scatters diagram of OLFML2B and Macrophage M1. **(C)** In the TIMER database, the correlation scatters diagram between OLFML2B and six kinds of immune cells. **(D)** The co-expression scatter map of OLFML2B and six types of macrophage markers. P < 0.05 indicates that it is statistically significant.

Because bladder cancer is an immunologically invasive cancer, we explored the relationship between OLFML2B and immune cell infiltration. First, the “CIBERSORT” algorithm was used to calculate the proportion of 22 types of immune cell in each sample in the TCGA-BLCA cohort (**Table S6**). Then, the Spearman correlations between OLFML2B expression and the immune cells were calculated (**Figure 8A**). There were positive correlations with macrophages M1 and macrophages M0 (cor > 0.3; P < 0.05) and negatively correlated with B memory cells and activated dendritic cells (cor <-0.3; P < 0.05). We drew a scatter plot of the relationships between OLFML2B and macrophages M1 as an example (**Figure 8B**). Then the relationships between OLFML2B and six types of immune cell were queried using the web tool “TIMER”. We found that the correlation between OLFML2B and macrophage was the highest (cor = 0.432; P = 4.77E-18; **Figure 8C**). Because both analyses showed that OLFML2B was positively correlated with macrophage content, we input cancer-related macrophage markers into the web tool “CellMarker” and obtained experimentally verified markers: CD14, CD68, CD163, CSF1R, ITGAM, and MRC1. The correlation scatter diagrams (**Figure 8D**) between OLFML2B and these six genes were drawn using the TIMER database. Except for low correlations with CD68 (cor = 0.378; P < 0.001), there were high correlations with the other five markers (cor >0.65; P < 0.001).

### OLFML2B Is Expressed at Higher Levels in BC Tissues and Promotes the Migration and Proliferation Ability of BC Cells

**Figure 9:**
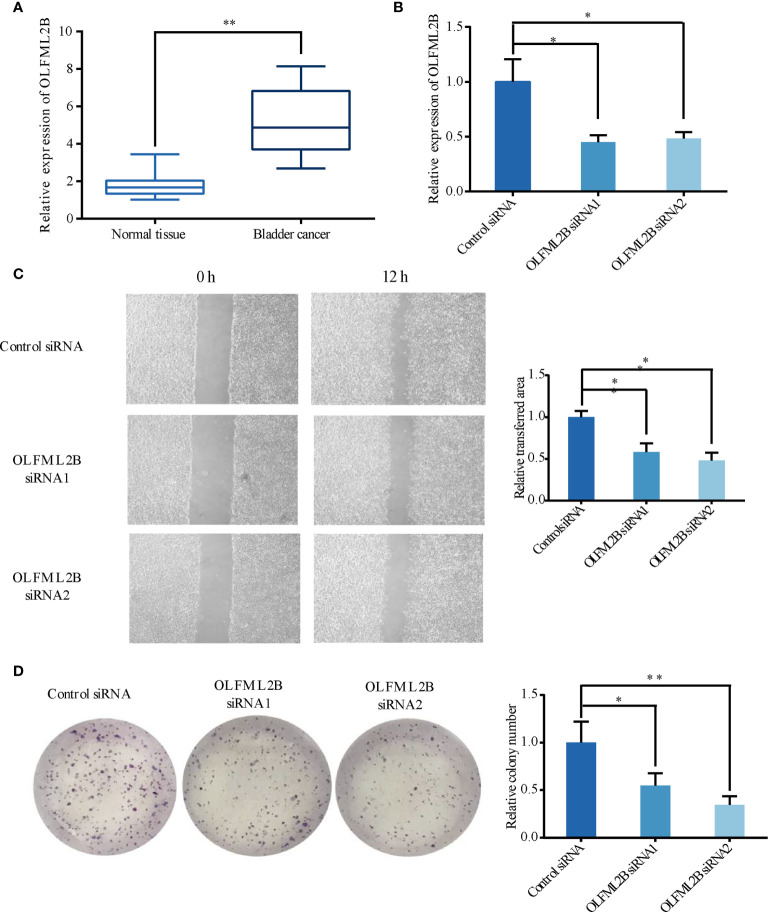
OLFML2B is expressed at higher levels in bladder cancer tissues and promotes bladder cancer cells’ migration and proliferation ability. **(A)** Higher expression levels of OLFML2B in BC tissues compared with corresponding normal tissues. **(B)** The relative mRNA levels of normal control and si-OLFML2B of T24 cells. **(C)** The Wound-healing assay results showed decreased migration ability of T24 cells treated with si-OLFML2B. **(D)**The results of the Colony formation assay showed decreased proliferation ability of T24 cells when treated with si-OLFML2B. The T-test is used for the differences between groups. “*” means P<0.05; “**” means P<0.01.

A total of 11 pairs of human BC tissues and their corresponding normal tissues were analyzed for OLFML2B expression by RT-qPCR. The results showed that OLFML2B was overexpressed in patients with bladder cancer (**Figure 9A**). Then, we used small interference RNA to reduce the expression level of OLFML2B in bladder cancer cell line T24 (**Figure 9B**). The wound-healing assay showed that the migration ability of cells decreased significantly after OLFML2B gene knockdown (**Figure 9C**). The clone formation assay showed that the proliferation ability of cells decreased significantly after OLFML2B gene knockdown (**Figure 9D**).

## Discussion

Bladder cancer is a disease with complex molecular characteristics, as well as high morbidity and mortality. Mining robust molecular markers will help to stratify the risk of patients to facilitate individualized treatment. In this study, Through the screening of genome-wide prognostic markers, we found OLFML2B with robust prognostic ability in multiple cohorts. Kaplan–Meier analysis of OLFML2B in seven cohorts showed that the prognosis of patients with significantly higher expression levels was worse.

OLFML2B successfully predicted the prognosis of patients with bladder cancer in seven cohorts. We downloaded transcriptome and clinical data of TCGA, GSE13507, E-MTAB-1803, GSE32548, and GSE32894 from multiple databases. Kaplan-Meier analysis of all genes showed that OLFML2B had a very stable prognostic ability in these five cohorts, and the prognosis was poor in the case of high expression of OLFML2B. The result was also confirmed by ROC curve, univariate Cox, and multivariate Cox. Finally, in two independent cohorts (GSE31684 and GSE48075), we successfully verified the prognostic ability of OLFML2B. The seven cohorts used in this study come from GEO, TCGA, and ArrayExpress databases, which are the three largest transcriptome databases at present, with real and reliable data sources and high authority. The sample sources of the seven cohorts are geographically diverse, three from North America, three from Europe and one from Asia, suggesting that this gene is applicable in a wide geographic area. The scale of our research is polycentric, cross-regional, and large-scale, suggesting that our results are authentic and reliable.

Olfactomedin-like 2B (OLFML2B) is an extracellular matrix protein containing the olfactomedin (OLF) domain, also known as photomedin-2 (28). Proteins containing OLF domains may participate in neurogenesis, intercellular adhesion, and tumorigenesis (29). OLFML2B is rich in Ser/Thr in the region upstream of the OLF domain, which is a difference from other OLF family proteins (30). OLFML2B is expressed in the retina and many other tissues (31). One of the important biological functions of OLFML2B is its ability to bind chondroitin sulphate-E and heparin selectively to regulate the binding of CS-E to growth factors (32). OLFML2B is a key factor in the perineural infiltration-related protein network of head and neck squamous cell carcinoma (33). Patients with high expression of OLFML2B had poorer prognosis in gastric cancer (34). In this study, we found that expression levels of OLFML2B increased with increased stage and grade, suggesting that OLFML2B promotes the progression of bladder cancer and increases the risk of invasion and deterioration of bladder cancer. OLFML2B is a potential oncogene of bladder cancer

OLFML2B also showed a robust prognostic ability in Kaplan-Meier analysis of pan-cancer. The prognosis of patients with high expression of OLFML2B was poor in eleven cancers including bladder cancer, which indicates that OLFML2B mainly plays a role in promoting cancer. OLFML2B has strong robustness in pan-cancer, which is very rare in gene markers. The prognostic ability of OLFML2B was only reported in gastric cancer (34), so it has great research potential in other cancers.

We sorted out 30 reported prognostic markers related to the expression level of bladder cancer from Mitra’s review (5) and then performed Kaplan–Meier analysis in seven bladder cancer cohorts. Among the 30 prognostic markers, BIRC5, IL6, MMP2, MMP9, had the most robust prognostic ability, which was successfully verified in four cohorts. BIRC5 (survivin), a member of the apoptosis inhibitor family, was associated with high specific mortality rate in 226 bladder cancer patients (35). Interleukin 6 (IL6) is a cytokine associated with poor prognosis of many cancers. It was reported that the expression of plasma IL6 in patients with bladder cancer before surgery is an independent predictor of disease-specific survival (36). Both MMP2 and MMP9 belong to the family of metalloproteinases. It was reported that the 5-year survival rate of MMP-2 positive cases was significantly lower than that of MMP-2 negative cases using immunohistochemical staining of 54 bladder cancer samples (37). MMP9 is associated with high-grade and distant metastasis of bladder cancer (38). After comparing the overall results of the two groups of genes, the proportion of successful verification of risk genes was higher than that of protective genes, while the opposite proportion was lower than that of protective genes. These two indicators suggest that risk genes are more stable in terms of prognostic ability than are protective genes, and OLFML2B happens to be a risk gene.

OLFML2B may be involved in the crosstalk between bladder cancer cells and macrophages. In the enrichment analysis of OLFML2B co-expression genes, we found that these genes were related to the tumor microenvironment, suggesting that OLFML2B may play an important role in the tumor microenvironment of bladder cancer. Bladder cancer has strong immune characteristics, and the use of checkpoint inhibitors are promising treatments for it (39). Therefore, we explored the relationship between OLFML2B and immune cells. The results of CIBERSORT and TIMER analysis showed that there was a positive correlation between OLFML2B and infiltration levels of macrophage. OLFML2B was highly co-expressed with tumor-associated macrophage markers such as CD14, CD163, CSF1R, ITGAM, and MRC1. Macrophages are highly plastic cells, and when they accumulate around the tumor, we call them tumor-associated macrophages (TAM) (40). TAM is an inhibitory immune cell that can receive tumor-derived signals to inhibit the infiltration of CD8+ T cell around tumor cells (23). TAM can also interact with extracellular matrix to promote cancer cell proliferation and invasion (41). We speculate that bladder cancer cells may secrete OLFML2B into the extracellular matrix and interact with TAM markers to guide TAM to work for itself. OLFML2B may be an immune target for TAM-related therapy.

Our experiments showed that OLFML2B was overexpressed in cancer tissues, and si-OLFML2B could significantly reduce the migration and proliferation of bladder cancer cell lines. These results suggest that OLFML2B is a potential marker and therapeutic target for patients with bladder cancer. However, our study has some limitations. First of all, we conducted genome-wide multiple hypothesis tests, but we did not correct the P-value because we did not find a suitable correction method and corresponding reference support. Secondly, more bladder cancer cohorts are needed to prove the possibility of OLFML2B as a marker, and more in-depth mechanism experiments are needed to prove the carcinogenicity of OLFML2B.

## Conclusions

In summary, OLFML2B successfully predicted bladder cancer prognosis in multiple cohorts with strong robustness and more potently than other reported genes. Our research is by far the largest cohort of studies on the prognosis of a single gene in bladder cancer and has important clinical significance. OLFML2B may be involved in the crosstalk between bladder cancer cells and tumor-associated macrophages, and is a potential immune therapeutic target. OLFML2B is a new prognostic marker for the individualized treatment of bladder cancer in the future.

## Data Availability Statement

Publicly available datasets were analyzed in this study. These data can be found here: TCGA_BLCA cohort come from TCGA database, https://www.cancer.gov/tcga;

The GSE13507, GSE31684, GSE32548, GSE32894, and GSE48075 cohorts come from GEO database, https://www.ncbi.nlm.nih.gov/gds/?term=;

The E-MTAB-1803 cohort come from ArrayExpress database; https://www.ebi.ac.uk/arrayexpress/.

## Author Contributions

DS conceived the study. JL and TL performed the bioinformatics analyses. JY downloaded and processed the data. JL wrote the manuscript. YZ and MY critically revised the article for essential intellectual content. All authors contributed to the article and approved the submitted version.

## Conflict of Interest

The authors declare that the research was conducted in the absence of any commercial or financial relationships that could be construed as a potential conflict of interest.

## Publisher’s Note

All claims expressed in this article are solely those of the authors and do not necessarily represent those of their affiliated organizations, or those of the publisher, the editors and the reviewers. Any product that may be evaluated in this article, or claim that may be made by its manufacturer, is not guaranteed or endorsed by the publisher.

## Supplementary Material

Supplementary Figure 1 Kaplan-Meier of OLFML2B in pan-cancer.

Supplementary Table 1 The specific information of cohorts.

Supplementary Table 2 The reported prognostic of bladder cancer.

Supplementary Table 3 The clinical information of patients in local hospital.

Supplementary Table 4 The result of Kaplan-Meier analysis.

Supplementary Table 5 The venn result of the five cohorts.

Supplementary Table 6 The immune cells infiltration level.

## Abbreviations

TCGA, The Cancer Genome Atlas; GEO, Gene Expression Omnibus; ROC, Receiver operating characteristic; AUC, Area under the curve; TAM, Tumor-associated macrophage.
