# Supplementary figures and images for: OLFML2B Is a Robust Prognostic Biomarker in Bladder Cancer Through Genome-Wide Screening: A Study Based on Seven Cohorts
Source: Front Oncol. 2021 Nov 15;11:650678. doi: 10.3389/fonc.2021.650678 (PMC8634430; doi:10.3389/fonc.2021.650678)

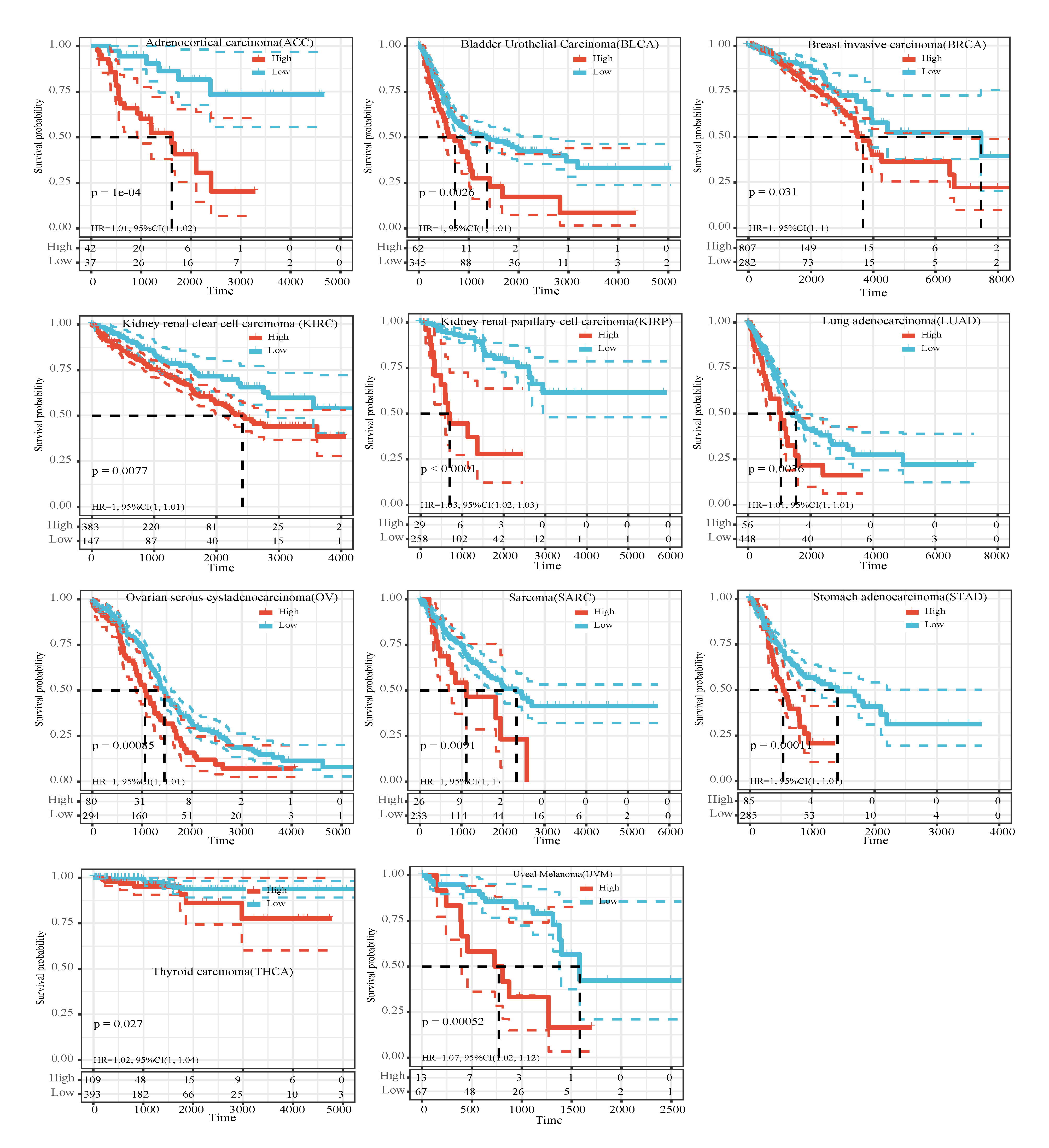

Supplement: Supplementary Figure 1 — Kaplan-Meier of OLFML2B in pan-cancer. [file Image_1.tif]
